# Circulating tumor DNA in lung cancer immunotherapy: prognostic marker today, predictive tool tomorrow?

**DOI:** 10.3389/fimmu.2026.1911583

**Published:** 2026-08-20

**Authors:** Yan Li, Guanyu Lu, Wei Song, Jin Lu, Xiangliang Liu, Jiuwei Cui

**Affiliations:** Cancer Center, First Hospital of Jilin University, Changchun, Jilin, China

**Keywords:** circulating tumor DNA, immune checkpoint inhibitors, lung cancer, minimal residual disease, predictive biomarker, prognostic biomarker, response-associated biomarker

## Abstract

Circulating tumor DNA (ctDNA) is emerging as a minimally invasive biomarker for risk stratification and treatment-response assessment in lung cancer immunotherapy, but its routine use for treatment selection has not been established. This review critically evaluates evidence across neoadjuvant, adjuvant, consolidation after definitive chemoradiotherapy, and advanced/metastatic settings, with emphasis on serial sampling and on the distinction among prognostic, response-associated, and predictive roles. Across disease stages, baseline or post-definitive-treatment ctDNA detectability consistently identifies patients at increased risk of recurrence or death and is therefore principally prognostic. Early on-treatment decline or clearance frequently precedes radiographic change and is associated with pathological response, progression-free survival, and overall survival, supporting ctDNA as a response-associated biomarker. By contrast, evidence that ctDNA identifies differential benefit from a specific immunotherapy remains limited, because most analyses are single-arm and/or retrospective, formal treatment-by-biomarker interaction tests are uncommon, and prospective ctDNA-guided trials have not yet demonstrated clinical utility. Nevertheless, ctDNA dynamics provide a biologically and clinically coherent framework for future risk-adapted strategies, including enrichment of molecular residual disease-positive patients, early identification of resistance, and prospective testing of treatment escalation, de-escalation, or duration. Translation into routine care will require harmonized assays and sampling time points, improved sensitivity at low disease burden, control of clonal hematopoiesis, and randomized interventional validation. Thus, ctDNA currently functions mainly as a prognostic and response-associated biomarker in lung cancer immunotherapy, while its predictive, decision-defining role remains an important but investigational objective.

## 1 Introduction

Immune checkpoint inhibitors (ICIs), alone or combined with chemotherapy and/or radiotherapy, are now integral to lung cancer management across the disease continuum, from curative-intent treatment of resectable disease to long-term disease control in advanced settings. However, clinical benefit is heterogeneous (1, 2). Some patients achieve durable remission, while others show primary resistance, relapse soon after definitive therapy, or experience toxicity with little clinical gain. This heterogeneity creates a central clinical challenge: how to identify patients who are most likely to benefit from ICI-based therapy and how to tailor treatment intensity and duration.

In lung cancer, tissue-based biomarkers only partly address this need. Programmed death-ligand 1 (PD-L1) expression correlates imperfectly with benefit, varies across tumor regions, and can change over time or after treatment. Tumor mutational burden (TMB) captures one aspect of immunogenicity, but it rarely performs well as a standalone predictor and is complicated by assay differences and sampling limitations (3–5). More fundamentally, a single biopsy is a static snapshot of a tumor that is spatially heterogeneous and constantly evolving; it does not reliably answer whether the current therapy is working right now, or whether it is likely to keep working.

Radiographic assessment in lung cancer has related limitations. Size-based response criteria often lag behind molecular and immune changes, and immunotherapy introduces interpretive challenges such as pseudoprogression and delayed response (6). Consequently, ineffective therapy may be continued unnecessarily, whereas patients with durable benefit may receive prolonged treatment with avoidable toxicity and cost because no validated dynamic biomarker defines response or optimal duration.

In this lung cancer-specific context, ctDNA offers a complementary approach (7–10). Because ctDNA reflects tumor burden and genomic heterogeneity, serial measurement can provide a real-time view of clonal dynamics under therapeutic pressure (11, 12). Conceptually, it makes sense that a rapid decline or clearance of ctDNA could serve as an early molecular signal of response-often before radiographic change-whereas rising ctDNA or re-emergent variants may indicate molecular progression or relapse before it becomes clinically obvious (12–17). In the minimal residual disease (MRD) setting after surgery or definitive chemoradiotherapy, ctDNA positivity can flag patients at high recurrence risk even when imaging remains negative. These potential advantages must be interpreted in light of variable tumor shedding, limited sensitivity at low disease burden, and false-positive signals from clonal hematopoiesis.

Throughout this review, we use three terms according to their conventional evidence requirements. A prognostic biomarker is associated with an outcome irrespective of the treatment received. A predictive biomarker identifies differential benefit from a specific therapy and is most convincingly supported by a statistically tested treatment-by-biomarker interaction in a randomized comparison. A response-associated biomarker correlates with response or outcome within a treated arm but, without an appropriate comparator and interaction analysis, does not establish treatment-specific benefit. We therefore reserve predictive for treatment-selective evidence, use response-associated for within-arm ctDNA-response correlations, and use prognostic for treatment-independent risk stratification.

**Figure 1:**
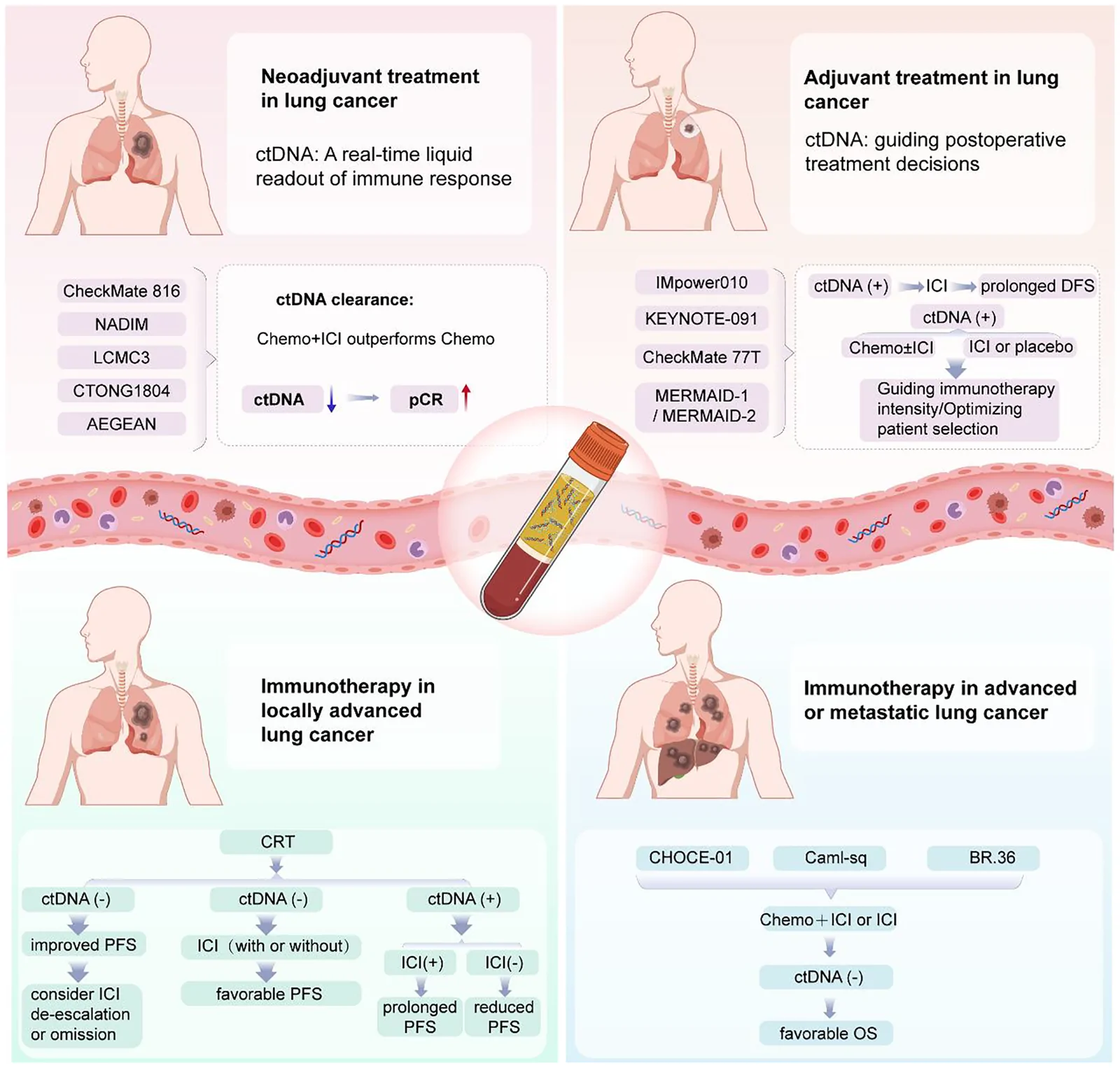
Prognostic, response-associated, and exploratory predictive evidence for ctDNA across lung cancer immunotherapy settings. In the neoadjuvant setting, early ctDNA decline or clearance is associated with pathological complete response (pCR) or major pathological response (MPR) and is response-associated, not proof of treatment-specific prediction. In the adjuvant setting, postoperative ctDNA mainly stratifies recurrence risk; differential immunotherapy benefit remains exploratory. For locally advanced disease, post-chemoradiotherapy ctDNA is prognostic, whereas comparisons of consolidation immune checkpoint inhibitor benefit across ctDNA groups provide exploratory predictive signals requiring prospective validation. In advanced/metastatic NSCLC, on-treatment ctDNA clearance is response-associated and prognostic; most studies have not demonstrated a treatment-by-biomarker interaction. The trials shown in image contribute different levels of evidence. CheckMate 816 was a randomized phase III trial of neoadjuvant nivolumab plus chemotherapy versus chemotherapy alone; ctDNA clearance occurred more frequently with nivolumab, but its association with pCR was evaluated after therapy and therefore supports a pharmacodynamic, response-associated role rather than pretreatment prediction (18). NADIM I/II evaluated perioperative nivolumab plus chemotherapy in resectable stage III NSCLC; very low or cleared ctDNA after neoadjuvant treatment was associated with MPR and longer survival, but the biomarker analyses were single-arm or exploratory (19, 20). LCMC3, CTONG1804, and AEGEAN reported concordant associations between perioperative ctDNA decline or clearance and pathological response, while CheckMate 77T extended longitudinal assessment into the adjuvant phase (21–26). In the adjuvant setting, IMpower010 and KEYNOTE-091 used postoperative ctDNA primarily to stratify recurrence risk, and treatment-selective utility remains unvalidated (27–29). In locally advanced and metastatic disease, Moding et al., CHOICE-01, CameL-sq, and BR.36 showed that post-treatment detectability or early ctDNA kinetics stratified risk or response, yet none prospectively used ctDNA to assign immunotherapy (11, 30–32).

These distinctions define both the scope and the evidentiary framework of this review. Most available data establish ctDNA as a prognostic marker: baseline or post-treatment detectability consistently stratifies risk. The more clinically actionable, but less established, objective is true prediction and decision support, defined here as using ctDNA to select patients for immunotherapy or to guide initiation, intensification, de-escalation, or discontinuation in ways that improve outcomes. Early ctDNA dynamics are often associated with response and survival, but association alone does not demonstrate treatment-specific benefit or clinical utility. Accordingly, general ctDNA biology and analytical principles are discussed only insofar as they determine interpretation in lung cancer immunotherapy. We then appraise evidence across four settings: neoadjuvant, adjuvant, consolidation after definitive chemoradiotherapy for unresectable stage III disease, and advanced disease (**Figure 1**), before addressing implementation barriers and priorities for prospective ctDNA-guided trials.

## 2 Lung cancer-specific biological rationale and technical framework for prognostic, response-associated, and candidate predictive ctDNA biomarkers

### 2.1 Biological basis

#### 2.1.1 Association of ctDNA with tumor burden and clonal evolution

In lung cancer, plasma ctDNA levels are influenced by the burden, turnover, and vascular accessibility of tumor deposits and can therefore serve as a molecular surrogate of disease burden (33). Serial profiling can also capture changes in clonal composition. Under the selective pressure of immunotherapy, contraction of treatment-sensitive clones is accompanied by a decline in ctDNA carrying their characteristic variants. Conversely, expansion of resistant subclones may increase the variant allele frequency of specific alterations and signal the outgrowth of resistant populations (34). Longitudinal ctDNA profiling can therefore complement imaging by tracking molecular tumor evolution during lung cancer immunotherapy (35).

#### 2.1.2 Characteristics of ctDNA dynamics in response to immunotherapy

In patients with lung cancer who respond to immunotherapy, tumor regression is commonly accompanied by a marked decrease, and sometimes clearance, of ctDNA, a pattern often termed molecular response (36). Early ctDNA decline is frequently associated with subsequent radiographic tumor shrinkage and may precede visible imaging changes, supporting its use as a response-associated readout. Conversely, persistent or rising ctDNA during treatment is associated with primary resistance or progressive disease (37).

Importantly, ctDNA dynamics offer a temporal advantage over conventional radiographic assessment. For example, in locally advanced NSCLC, a rise in ctDNA may precede radiographic progression by approximately four months. Thus, a decrease or clearance of ctDNA is regarded as a molecular hallmark of effective immunotherapy, whereas persistently elevated or rising ctDNA indicates a poor response. This pattern of molecular response is closely associated with both progression-free survival (PFS) and overall survival (OS), and supports the use of ctDNA as a valuable complementary biomarker for evaluating the efficacy of immunotherapy (38–40).

### 2.2 Key technologies and methodologies

#### 2.2.1 Detection architectures and representative platforms

At the highest level, mutation-based ctDNA assays use tumor-informed or tumor-agnostic architectures. Tumor-informed assays begin with somatic variants identified in an individual patient’s tumor and track a personalized set of variants, which generally improves sensitivity and specificity at low disease burden. Tumor-agnostic (plasma-only) assays do not require tissue genotyping and instead interrogate predefined genes or broader genomic features in plasma. Both architectures may be implemented using targeted sequencing. Cancer Personalized Profiling by Deep Sequencing (CAPP-Seq) uses cancer-specific hybrid-capture panels with integrated error suppression, while phased-variant methods can further improve sensitivity by requiring co-occurring variants on the same DNA molecule. Targeted Error Correction Sequencing (TEC-Seq) combines ultra-deep sequencing with molecular error correction and is useful when tissue is unavailable or the tumor genotype is unknown (41–43). Targeted Error Correction Sequencing (TEC-Seq) combines ultra-deep sequencing with molecular error correction and is useful when tissue is unavailable or the tumor genotype is unknown (44, 45).

Analytically, these architectures can be implemented using allele-specific quantitative polymerase chain reaction (qPCR), droplet digital PCR (ddPCR), or targeted next-generation sequencing (NGS). PCR-based methods provide rapid, highly sensitive quantification of a small number of predefined variants and are therefore well suited to serial tracking when the target is known (46, 47). Targeted NGS uses amplicon or hybrid-capture panels and may incorporate unique molecular identifiers (UMIs), duplex consensus sequencing, integrated error suppression, or phased-variant tracking to suppress background noise and improve sensitivity at low tumor fraction (41–45). NGS provides greater breadth for monitoring multiple clones, but requires deeper sequencing, more complex bioinformatics, and assay-specific threshold calibration (48).

Beyond mutation counting, emerging plasma assays assess genome-wide copy-number alterations, methylation patterns, fragment lengths and end motifs, nucleosome footprints, or combinations of genomic and epigenomic signals. Low-pass whole-genome sequencing (WGS) can support copy-number and fragmentomic analyses without predefined variants, whereas methylation and fragmentomic approaches may improve detection in tumors with few trackable mutations. However, evidence linking these modalities to serial response assessment or treatment-by-biomarker interactions in lung cancer immunotherapy remains limited (45, 49, 50).

For the clinical use cases reviewed here, there is no single universally superior platform. Tumor-informed sequencing or ddPCR is advantageous for high-sensitivity MRD and known-variant tracking; broader plasma-only NGS is useful when tissue is unavailable or clonal evolution is of interest; and genome-wide or epigenomic assays remain investigational for immunotherapy-guided decisions. In all cases, analytical sensitivity, blood volume, turnaround time, false-positive control, and reproducibility at prespecified time points must match the intended clinical use (49, 51, 52).

#### 2.2.2 ctDNA readouts relevant to lung cancer immunotherapy

For lung cancer immunotherapy, ctDNA readouts can be organized as static baseline measures, dynamic kinetic parameters, and composite metrics. Baseline detectability, mutant-copy level, and variant allele frequency (VAF), including maximum VAF (maxVAF), primarily reflect tumor burden and overall risk (53–55), and are therefore generally prognostic rather than predictive of immunotherapy benefit. Dynamic indicators capture change over time, including ctDNA clearance (conversion from detectable to undetectable or below a prespecified threshold) and VAF kinetics, such as the relative change in mean mutant VAF (mmVAF) (56). Composite indices summarize these shifts across variants; MinerVa-Delta, for example, integrates the on-treatment change in mutation burden into a single quantitative score (57).

Among these, molecular response-typically defined as ctDNA clearance or a marked decline (often >50%) after a set number of cycles-appears most clinically informative and is repeatedly associated with longer PFS and OS (57). The flip side is molecular progression: rising ctDNA can precede radiographic progression, likely reflecting emerging resistance, and it generally portends poor outcomes (58).

Within the same monitoring framework, mmVAF has been used to probe clonal structure. In locally advanced NSCLC treated with chemoradiotherapy plus immunotherapy, a higher baseline mmVAF has been interpreted as a signal of lower heterogeneity and higher clonality, potentially enriching for shared clonal neoantigens that are more readily targeted by activated immune cells (56). Consistent with that idea, patients with high baseline mmVAF often show rapid ctDNA declines and better outcomes, whereas low baseline mmVAF (suggesting greater heterogeneity) and absent early ctDNA decline tend to align with immune escape and reduced benefit (56). MinerVa-Delta offers another angle: in first-line immunotherapy for advanced lung squamous cell carcinoma, residual ctDNA ≥30% after two cycles was used to define molecular non-response and, notably, outperformed RECIST-based imaging for predicting efficacy and survival-especially among patients labeled as stable disease (SD) (57).

It is also increasingly clear that ctDNA and imaging can complement each other (59). Among radiographic SD cases, molecular clearance by ctDNA often corresponds to meaningful clinical benefit, with outcomes closer to partial response, whereas SD without ctDNA decline tends to resemble progressive disease (57). In that sense, integrating ctDNA kinetics with radiology may help correct the blind spots of imaging alone and sharpen response stratification under immunotherapy.

#### 2.2.3 Sample processing and quality control in lung cancer immunotherapy studies

Reliable longitudinal ctDNA assessment in lung cancer depends on standardized pre-analytical handling and quality control. Plasma is preferred over serum because clotting can release background DNA from blood cells, dilute the tumor signal, and reduce mutation-detection specificity (60, 61). Samples should be processed promptly to separate plasma and stored at low temperature to limit leukocyte lysis and genomic DNA contamination. Timing also matters. In neoadjuvant/adjuvant settings, serial sampling at baseline (pre-surgery), post-surgery, and defined treatment time points is particularly useful for MRD assessment and for gauging adjuvant efficacy (18, 62). In advanced disease, early kinetics-often assessed about 2–6 weeks after treatment initiation-appear especially informative for response prediction (63).

A key confounder is clonal hematopoiesis (CH). Age-associated mutations in genes such as DNMT3A, TET2, and JAK2 can arise from expanded hematopoietic clones and shed into circulation independent of tumor, creating false-positive “ctDNA” signals (64). A practical mitigation is parallel sequencing of matched peripheral blood leukocyte DNA, followed by bioinformatic filtering to remove CH-derived variants. With standardized processing, appropriate sampling schedules, and CH-aware interpretation, ctDNA testing becomes substantially more robust for response monitoring and exploratory treatment-selection research in lung cancer.

## 3 Prognostic, response-associated, and exploratory predictive value of ctDNA across immunotherapy treatment settings

### 3.1 Prognostic and response-associated utility of ctDNA in neoadjuvant immunotherapy (immunotherapy combined with chemotherapy) for lung cancer

In resectable NSCLC, neoadjuvant immunotherapy combined with chemotherapy has become a major step forward. In this setting, dynamic ctDNA changes can provide a timelier and often more sensitive signal of treatment response than conventional radiographic imaging (65). In a prospective study by Yue et al., the relative change in mean VAF (RΔmean VAF) during treatment was associated with MPR and classified MPR status with 100% sensitivity and 91.7% accuracy, substantially outperforming computed tomography (CT)-based assessment. In practical terms, ctDNA kinetics may allow early identification of pathological responders, whereas persistent ctDNA positivity tends to be associated with limited pathological response (66).

Exploratory biomarker data from CheckMate 816 align with this concept. Patients receiving neoadjuvant nivolumab plus chemotherapy showed a higher ctDNA clearance rate (56%) than those treated with chemotherapy alone (34%). Importantly, across treatment arms, ctDNA clearance was strongly associated with a higher pathological complete response (pCR) rate (46% vs. 13%), supporting ctDNA clearance as a response-associated pharmacodynamic readout. Because clearance was evaluated after treatment and the response association was assessed across arms, these findings do not establish that ctDNA predicts incremental benefit from nivolumab (18).

Similar patterns are reported in NADIM I/II and LCMC3. In NADIM I/II, most patients who cleared ctDNA after neoadjuvant immunochemotherapy achieved MPR (19, 20). In LCMC3, which evaluated neoadjuvant atezolizumab monotherapy, patients with MPR experienced a significantly greater ctDNA reduction than non-responders, and the magnitude of decline correlated moderately with both pathological response and radiographic tumor shrinkage. This suggests ctDNA kinetics track genuine biological activity even when immunotherapy is given without chemotherapy (21, 22). Pooled analyses, including CTONG1804, further reinforce that conversion from ctDNA positivity to negativity is a robust marker of deep pathological response to immunochemotherapy. Patients maintaining ctDNA negativity across serial timepoints consistently achieved pCR/MPR, whereas those with any persistent ctDNA were largely non-responders (23).

ctDNA dynamics could also support prospective studies of neoadjuvant treatment duration. In NCT05157776, which compared different cycles of neoadjuvant immunochemotherapy, MRD dynamics based on ctDNA helped distinguish patients more likely to benefit from extended therapy. Those who cleared ctDNA early and sustained negativity achieved a pCR rate of 75%, compared with 0% among patients who never cleared ctDNA (24). Larger biomarker datasets, including AEGEAN, show similar trends, with higher preoperative ctDNA clearance and improved pCR/MPR rates in patients receiving durvalumab plus chemotherapy; conversely, failure to clear ctDNA was rarely followed by deep pathological response (25). These data support ctDNA kinetics as a response-associated pharmacodynamic readout, but they do not yet validate ctDNA-guided modification of neoadjuvant duration (67).

In summary, baseline ctDNA detectability in the neoadjuvant setting is principally prognostic, whereas on-treatment decline or clearance is response-associated with the depth of pathological response. These measures can identify molecular and pathological response before long-term survival endpoints mature, but current data do not establish a treatment-selective predictive biomarker without a treatment-by-ctDNA interaction.

### 3.2 Prognostic, response-associated, and exploratory predictive applications of ctDNA in adjuvant immunotherapy post-surgery for lung cancer

The introduction of adjuvant immunotherapy for resectable non-small cell lung cancer (NSCLC) has raised a central question: which patients truly need and are most likely to benefit from it? Circulating tumor DNA (ctDNA), as a sensitive marker of minimal residual disease (MRD), has been studied primarily for prognostic stratification and, more recently, as an enrichment variable in trials testing whether immunotherapy benefit differs by ctDNA status (68–71).

The phase III IMpower010 trial prospectively included ctDNA analysis in resected stage IB-IIIA NSCLC after adjuvant chemotherapy. Detectable ctDNA after surgery and chemotherapy was associated with significantly shorter disease-free survival (DFS) than undetectable ctDNA, regardless of treatment, confirming its prognostic role (28). Later analyses showed that ctDNA clearance after chemotherapy was associated with improved long-term DFS. Although a trend suggested greater atezolizumab benefit in ctDNA-positive, PD-L1-positive patients, the formal interaction test was not significant. Thus, ctDNA identifies a high-risk subgroup for risk-adapted trial design but is not a validated predictive biomarker for selecting adjuvant atezolizumab (27).

Exploratory analyses from KEYNOTE-091 further supported this role. Detectable ctDNA after surgery identified patients who derived clinically meaningful DFS improvement from adjuvant pembrolizumab, irrespective of PD-L1 expression (29). Similarly, in CheckMate 77T, patients with persistent ctDNA after neoadjuvant immunochemotherapy and surgery gained substantial event-free survival(EFS) benefit from adjuvant nivolumab. Conversion from ctDNA-positive to negative during treatment provided direct evidence of molecular remission, supporting a response-associated role for on-treatment ctDNA; these analyses do not establish a treatment-by-biomarker interaction (26).

The TRACERx study provided the biological rationale for this risk-adapted approach. In early-stage NSCLC, postoperative ctDNA detection reflected persistent residual clones with metastatic potential and was strongly associated with recurrence and death, defining a molecular residual disease population. Although TRACERx did not evaluate immunotherapy, it delineates a clear high-risk group in whom adjuvant treatment may be most rationally directed (72–75).

New trials have been designed around ctDNA-guided strategies. MERMAID-1 and MERMAID-2 used tumor-informed ctDNA assays to direct postoperative durvalumab in MRD-positive patients without radiographic recurrence. These studies evaluated ctDNA dynamics-clearance, persistence, or re-emergence-as candidate markers for treatment selection. MERMAID-2 specifically triggered early immunotherapy upon molecular relapse (ctDNA conversion from negative to positive), while observing MRD-negative patients (76, 77). Because both trials terminated early and lack definitive efficacy results, they do not establish predictive validity; nevertheless, their designs mark a methodological shift from prognostic enrichment toward prospective testing of ctDNA as a treatment-selection marker in adjuvant NSCLC (78–80).

While current evidence remains exploratory and insufficient to use ctDNA as the sole determinant for immunotherapy, it already informs clinical reasoning. ctDNA positivity signals residual disease, high recurrence risk, and a subgroup with potential for benefit from treatment intensification. Conversely, ctDNA negativity suggests low tumor burden and supports a more individualized discussion on whether-and how intensively-adjuvant immunotherapy is warranted (81).

### 3.3 Prognostic, response-associated, and exploratory predictive application of ctDNA in consolidation immunotherapy after chemoradiotherapy

Although the PACIFIC regimen, which combines chemoradiotherapy (CRT) with consolidation immunotherapy, is now the standard approach for locally advanced unresectable NSCLC, outcomes remain heterogeneous. Some patients appear to gain little additional benefit from prolonged immune checkpoint inhibitor (ICI) treatment, while others relapse quickly despite therapy. This variability highlights the need for biomarkers, particularly MRD, to guide treatment intensity and duration.

Emerging data suggest ctDNA-based MRD assessment could support prospective testing of consolidation intensity and duration. A subset of patients may achieve deep molecular remission with CRT alone. For example, Pan et al. reported that about 20% of patients remained longitudinally ctDNA-negative throughout CRT and had excellent long-term survival, providing a rationale for testing de-escalated consolidation in this group (82). In locally advanced NSCLC, Moding et al. provided retrospective exploratory evidence linking ctDNA status to differential ICI benefit. Patients with undetectable ctDNA after CRT had favorable outcomes regardless of consolidation ICI, and a treatment-related death in this group illustrated the potential cost of overtreatment. By contrast, among patients with detectable post-CRT ctDNA, consolidation ICI was associated with improved outcomes. Early ctDNA kinetics during consolidation further distinguished response-associated subgroups (11).

The timing of MRD assessment also matters. Yang et al. suggested that measuring ctDNA one month after CRT completion offers strong prognostic separation and may represent a practical decision point for initiating consolidation (83). ctDNA kinetics may also help refine treatment duration. In BTCRC LUN 16-081, patients who achieved sustained ctDNA negativity had excellent outcomes despite a median consolidation duration of only 4.5 months, which raises a reasonable question about whether a full one-year course is necessary for molecular responders. By contrast, persistent or rising ctDNA was associated with poor outcomes and may indicate a need for intensified strategies (84).

**Figure 2:**
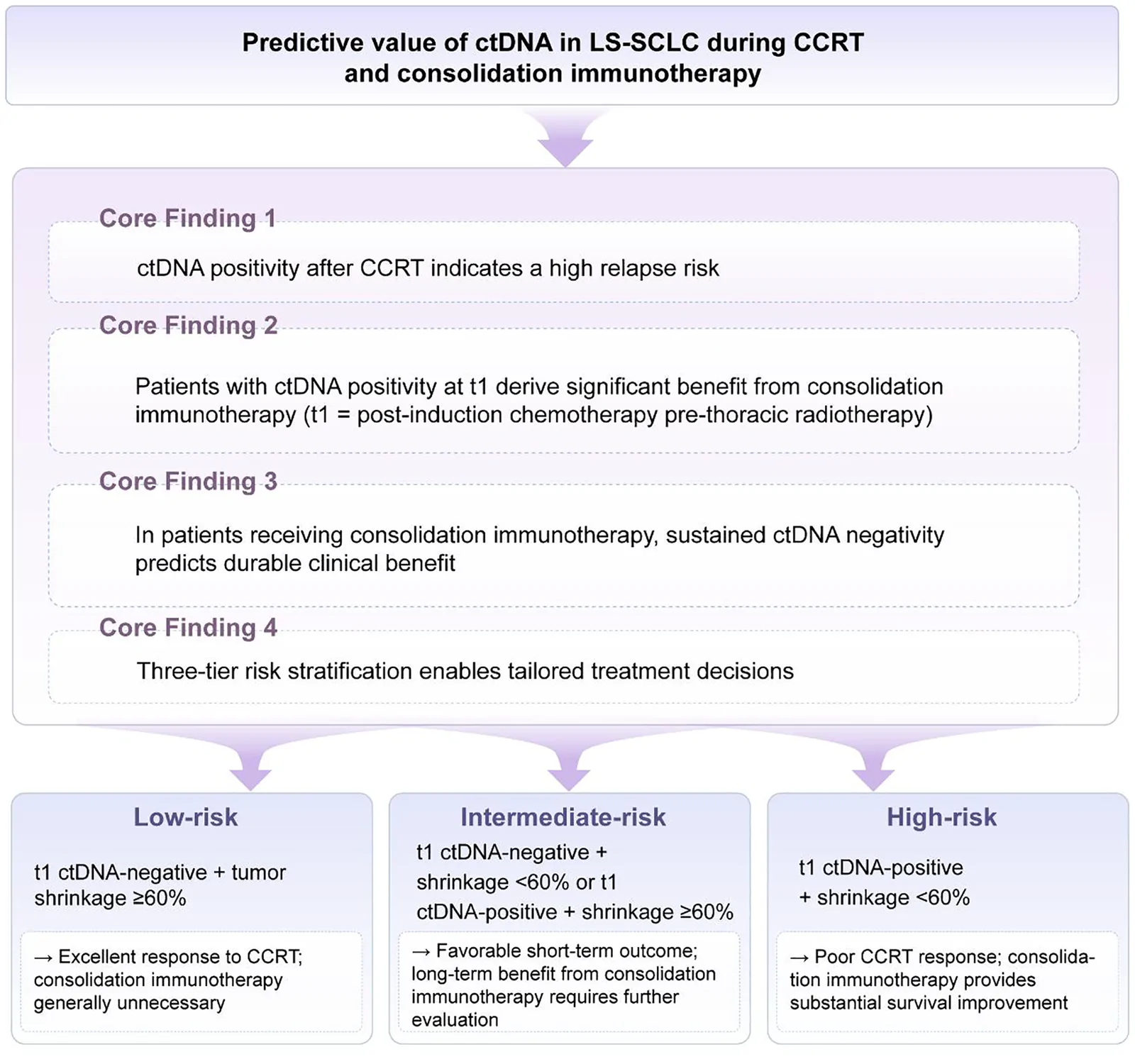
Prognostic, response-associated, and exploratory predictive utility of ctDNA in limited-stage small-cell lung cancer (LS-SCLC) receiving chemoradiotherapy with or without consolidation immunotherapy. This figure specifically summarizes the LS-SCLC cohort reported by Yang et al. (85), separate from the preceding NSCLC evidence. Post-CCRT ctDNA positivity was prognostic of recurrence. Comparison of consolidation ICI versus CCRT alone across ctDNA-defined groups suggested exploratory predictive value, most evident in the high-risk group defined by t1 ctDNA positivity and tumor shrinkage <60%. Sustained ctDNA negativity during consolidation ICI was response-associated with favorable outcomes. The three-tier model combined t1 ctDNA status and radiographic tumor shrinkage: low risk, t1 ctDNA-negative with shrinkage ≥60%; intermediate risk, t1 ctDNA-negative with shrinkage <60% or t1 ctDNA-positive with shrinkage ≥60%; and high risk, t1 ctDNA-positive with shrinkage <60%.

To extend this discussion beyond NSCLC, Yang et al. evaluated a distinct cohort of 144 patients with limited-stage small-cell lung cancer (LS-SCLC), including 100 treated with concurrent chemoradiotherapy (CCRT) alone and 44 who received consolidation serplulimab. Post-CCRT ctDNA positivity was prognostic, whereas comparisons of consolidation ICI benefit across ctDNA-defined groups provided exploratory predictive evidence. Integrating ctDNA status at the first post-CCRT landmark (t1) with radiographic tumor shrinkage yielded a three-tier model; the high-risk group (t1 ctDNA-positive with <60% shrinkage) appeared to derive the greatest benefit from consolidation ICI (85) (**Figure 2**). During consolidation, serial ctDNA monitoring can provide real-time feedback: sustained negativity suggests ongoing benefit, whereas re-emergence may signal evolving resistance and the need to reconsider strategy.

Taken together, the NSCLC and LS-SCLC data support complementary but distinct roles: post-chemoradiotherapy ctDNA is prognostic, on-treatment kinetics are response-associated, and differential ICI benefit across ctDNA-defined groups constitutes exploratory predictive evidence. Prospective trials with prespecified interaction testing are required before ctDNA can determine who should receive consolidation immunotherapy or its optimal duration.

### 3.4 Prognostic and response-associated roles of ctDNA and its exploratory predictive potential in advanced-stage lung cancer immunotherapy

With ICIs now established as standard first-line therapy for advanced NSCLC, the key clinical question has shifted from whether to use immunotherapy to which patients truly benefit from it. Conventional radiographic assessments are often delayed and sometimes non-informative, and tissue-based biomarkers such as PD-L1 and TMB have limited predictive accuracy. In contrast, ctDNA captures circulating tumor burden and clonal evolution in real time, providing dynamic molecular information early during treatment and emerging as a particularly informative biomarker system for predicting ICI efficacy in advanced NSCLC (86).

Several first-line immunochemotherapy studies have laid the foundation for integrating ctDNA into predictive models. In CHOICE-01, Wang et al. showed that PD-1 inhibitor plus chemotherapy significantly improved PFS and OS (13), and subsequent work in *Signal Transduction and Targeted Therapy* demonstrated that combining baseline ctDNA levels, mutational features, and immune microenvironment characteristics improved identification of patients who derive the greatest benefit (30). In squamous NSCLC, the CameL-sq study led by Zhou et al. similarly confirmed the survival advantage of immunochemotherapy and suggested that ctDNA clearance has early predictive value for treatment response (31).

Prospective longitudinal studies further highlight ctDNA as an early and sensitive indicator of efficacy. In the first stage of BR.36 (NCT04093167), which systematically evaluated ctDNA in first-line immunotherapy, 50 patients with advanced NSCLC received pembrolizumab monotherapy. Complete ctDNA clearance by the third treatment cycle, defined as a molecular response, strongly predicted long-term survival, with median OS not reached in responders compared with 7.23 months in non-responders, and outperformed contemporaneous radiographic assessment (32). In a larger validation cohort analyzed with TEC-Seq, molecular response, defined as complete clearance of ctDNA signal, correlated closely with PFS and OS, independent of PD-L1 expression and treatment regimen (63). Notably, in patients classified as having stable disease by RECIST, ctDNA molecular response identified true beneficiaries 4 to 6 weeks earlier than imaging. Together, these data support ctDNA as a robust response-associated biomarker during immunotherapy, but not as proof of treatment-specific predictive benefit.

Across studies in advanced lung cancer immunotherapy, current evidence points to three potential applications for ctDNA, each at a different level of maturity. First, baseline or early on-treatment ctDNA can refine prognostic stratification, but it should not by itself determine regimen intensity. Second, serial clearance, sustained decline, re-emergence, or rise provides a response-associated measure of efficacy and resistance that may precede radiographic change by 1–4 months; whether changing treatment on this basis improves outcomes remains unproven. Third, among patients with radiographic stable disease, molecular response may help distinguish biological benefit from occult progression and may complement benefit-risk assessment when immune-related toxicity complicates management. Even in these settings, ctDNA complements rather than replaces imaging and clinical assessment. Accordingly, ctDNA can inform hypotheses concerning treatment initiation, regimen selection, duration, de-escalation, or switching, but these decisions require prospective evaluation in ctDNA-guided trials before routine adoption (key studies are summarized in **Table 1**).

**Table 1:** Key studies supporting prognostic, response-associated, and exploratory predictive roles of ctDNA in lung cancer immunotherapy.

Study	Clinical scenario	Design & ctDNA-evaluable population	ctDNA strategy & sampling time points	Main ctDNA endpoints	Key associations with treatment outcomes	Evidence level/remarks
CheckMate 816 (18)	Resectable stage IB–IIIA NSCLC; neoadjuvant nivolumab + chemotherapy ×3 vs chemotherapy ×3	Phase III randomized trial; n=358 (exploratory ctDNA subset n=89)	Tumor-informed (ArcherDX Personalized Cancer Monitoring); C1D1, C3(pre-surgery)	ctDNA clearance (detectable→undetectable), ΔVAF	Higher ctDNA clearance with nivolumab + chemo vs chemo (≈56% vs 35%); patients with clearance had longer EFS/OS and higher pCR/MPR, with concordant directions of benefit	High quality RCT; ctDNA prespecified as exploratory endpoint; ctDNA evaluable rate limited
checkMate77T (26)	Resectable stage IIA–IIIB NSCLC; perioperative nivolumab + platinum-doublet ×4 vs placebo + chemotherapy ×4, followed by 1-year adjuvant nivolumab vs placebo	Phase III double-blind randomized trial; n=461 (ctDNA prespecified exploratory endpoint; ~190 with longitudinal ctDNA; neoadjuvant C1D1 ctDNA evaluable ≈176; clearance analysis: nivolumab n=76 vs placebo n=64)	Tumor-informed assay; samples at neoadjuvant C1D1 and end of neoadjuvant/pre-surgery; during adjuvant phase at C1D1, C4D1, C7D1, C13D1 and at radiographic relapse	Neoadjuvant ctDNA clearance (detectable → undetectable); ctDNA recurrence during adjuvant phase (negative → positive)	Neoadjuvant ctDNA clearance rate higher with nivolumab vs placebo (66% vs 38%); clearance associated with longer EFS and strongly correlated with pCR; molecular recurrence during adjuvant treatment less frequent with nivolumab (≈8% vs 20%)	High-quality RCT; ctDNA predefined as translational endpoint, but treatment intensity was not adapted according to ctDNA, serving mainly as strategy-generating evidence
AEGEAN (25)	Resectable stage II–IIIB NSCLC; perioperative durvalumab + platinum-chemotherapy ×4 followed by adjuvant durvalumab ×12 vs placebo+chemotherapy×4 followed by placebo	Phase III double-blind randomized trial; N = 802 (ctDNA prespecified translational endpoint; NEJM primary report did not provide detailed ctDNA sample numbers or full analyses)	Tumor-informed; baseline (pre-C1D1), before each of the 4 neoadjuvant cycles (C1D1, C2D1, C3D1, C4D1), at the pre-surgical assessment, and at a post-surgery landmark time point before initiation of adjuvant durvalumab/placebo, with additional longitudinal samples during postoperative follow-up in exploratory analyses	ctDNA clearance; early ΔVAF	Pre-surgical ctDNA clearance higher with durvalumab vs control (≈66% vs 41%); early (e.g. C2) clearance associated with higher pCR/MPR rates and a trend toward improved EFS	High-quality RCT; ctDNA predefined as translational endpoint, with detailed data mainly reported in subsequent translational/meeting reports rather than the primary NEJM paper
NADIM (20)	Resectable stage IIIA NSCLC; neoadjuvant nivolumab + chemotherapy ×3 followed by surgery and 1-year adjuvant nivolumab	Prospective single-arm phase II; n=46 ITT (ctDNA-evaluable: baseline n=43, post-neoadjuvant n=40)	Tumor-informed NGS; plasma at pre-neoadjuvant baseline and after neoadjuvant therapy (pre-surgery), with follow-up sampling in some patients	ctDNA clearance; limit of detection 0.1% MAF; baseline risk stratified at 1% MAF	Patients with ctDNA <0.1% or with ctDNA clearance after neoadjuvant therapy had significantly longer PFS/OS; ctDNA-based risk stratification outperformed RECIST response, TMB and PD-L1, suggesting perioperative nivolumab-chemo ctDNA level and clearance as strong prognostic/response-associated biomarkers	Prospective phase II; moderate sample size but rich longitudinal ctDNA; key evidence for ctDNA as a perioperative prognostic marker
IMpower010 (28, 87)	Completely resected stage IB–IIIA NSCLC; adjuvant platinum-based chemotherapy followed by 1-year atezolizumab vs best supportive care	Phase III randomized trial; ITT n=1005. ctDNA prespecified/exploratory translational cohort; ~600 patients with post-operative samples who received ≥1 cycle of chemotherapy	Tumor-informed MRD assay; blood drawn 4–10 weeks after surgery and before starting adjuvant therapy (main landmark time-point)	Post-operative/pre-adjuvant ctDNA positive vs negative; exploratory analyses of post-chemotherapy ctDNA clearance	Post-operative ctDNA positivity strongly associated with shorter DFS; atezolizumab significantly improved DFS in PD-L1–positive subgroups, but ctDNA status has not yet clearly defined differential immunotherapy benefit, indicating ctDNA is predominantly a prognostic rather than predictive marker at present	Large phase III RCT with prespecified/exploratory ctDNA translational work; first adjuvant IO phase III trial to demonstrate strong prognostic value of post-operative ctDNA MRD, but not yet used to select atezolizumab treatment
LCMC3(meeting/translational analyses)	Resectable stage IB–IIIB NSCLC; neoadjuvant atezolizumab ×2 cycles, optional adjuvant atezolizumab in some patients	Single-arm phase II; total n=181, ~106 included in ctDNA translational cohort forming the ctDNA-evaluable “resected efficacy population”	Tumor-informed assay (AVENIO Oncology Surveillance Test); plasma at baseline, after completion of neoadjuvant atezolizumab, and post-surgery	ctDNA clearance; ΔVAF	Decreases/clearance of ctDNA after neoadjuvant therapy associated with greater tumor shrinkage and higher MPR rates; post-surgery ctDNA negativity associated with numerically better DFS	Single-arm phase II with *post hoc* translational analysis; results reported mainly in conference abstracts, no full peer-reviewed ctDNA paper yet; ctDNA appears as a strong prognostic marker, but predictive value and ctDNA-guided strategies remain to be validated
Moding2020 (11)	Unresectable stage III NSCLC treated with CRT ± consolidation durvalumab	Retrospective cohort; 65 patients received definitive CRT, 28 continued durvalumab; all included in ctDNA analyses	Tumor-informed CAPP-Seq; plasma at baseline, after completion of CRT, and before cycles 1, 2 and 3 of consolidation IO (≈0, 6, 9–12 weeks) to track ctDNA dynamics	Post-CRT ctDNA status (negative vs positive); early on-treatment ctDNA dynamics (decline/clearance vs increase)	Post-CRT ctDNA negativity identified patients with excellent outcomes regardless of IO; among post-CRT ctDNA-positive patients, only those receiving IO had significantly improved failure-free survival; early ctDNA decline/clearance on IO predicted durable benefit, whereas rising ctDNA indicated primary resistance and early progression	High-quality translational study; first to systematically demonstrate that ctDNA dynamics predict benefit from consolidation IO after CRT in LA-NSCLC; ctDNA acts as both prognostic and predictive biomarker
CHOICE-01 (30)	Stage IV NSCLC; first-line PD-1 inhibitor (toripalimab) + chemotherapy vs chemotherapy alone	Phase III, double-blind randomized trial; 461 patients; biomarker cohort ~188 ctDNA-evaluable	Tumor-informed NGS; plasma at baseline and after 2 treatment cycles (C2D1)	Baseline blood TMB (bTMB); ctDNA abundance change (ΔVAF); ctDNA clearance rate	Low baseline ctDNA and early ctDNA decline/clearance were associated with significantly improved PFS/OS; early ctDNA increase identified a high-risk, treatment-resistant subgroup	One of the largest multi-omics translational analyses in Chinese first-line IO-chemo; ctDNA shown as a strong early predictor of benefit, though analyses remain exploratory
CameL-sq (31)	Stage IIIB–IV squamous NSCLC; camrelizumab + carboplatin/paclitaxel (4–6 cycles) followed by camrelizumab maintenance vs placebo + same chemotherapy	Phase III randomized double-blind trial; n=389 (exploratory ctDNA subset n=134)	Tumor-informed NGS; C0 (before initial treatment) and C2 (after two cycles)	ΔVAF; ctDNA clearance	Greater ΔVAF reduction and ctDNA clearance were independently associated with prolonged PFS/OS; ctDNA dynamics were a strong predictor of treatment effect with camrelizumab + chemotherapy	High-quality RCT; ctDNA predefined exploratory biomarker analysis supporting the predictive value of dynamic ctDNA monitoring in IO-chemo
BR36 (32)	Advanced NSCLC; first-line PD-1 monotherapy	Prospective phase II; 50 patients with multi-time-point ctDNA sampling	WBC-informed NGS (tumor-agnostic); plasma at C1D1, C2D1 and C3D1	maxMAF clearance (molecular response, mR)	mR showed high concordance with RECIST (sensitivity 82%, specificity 75%); mR associated with markedly longer OS and PFS; ctDNA response allowed early stratification of SD patients (SD + mR with durable benefit vs SD + molecular progression with early failure)	First prospective trial to formally validate the definition and optimal timing of ctDNA molecular response to immunotherapy in NSCLC; key proof-of-concept for ctDNA-guided IO strategies

## 4 Limitations in clinical application

The evidence reviewed above establishes substantial clinical validity for ctDNA-based risk stratification and response assessment in lung cancer immunotherapy; it does not yet establish clinical utility for treatment selection. Technical and analytical variability, biological limitations, and the current level of interventional evidence therefore preclude routine decision-defining use.

### 4.1 Technical and analytical limitations

The main technical challenges relate to detection sensitivity and lack of standardization. ctDNA is present at very low concentrations, particularly in patients with MRD after surgery or chemoradiotherapy, where tumor-derived DNA represents only a small fraction of total cell-free DNA. As a result, a negative ctDNA result cannot be interpreted as proof of complete disease eradication, which is especially relevant in adjuvant and consolidation immunotherapy settings (51, 88). Substantial variability also exists among platforms with respect to pre-analytical handling (such as collection tubes and processing time), sequencing depth, error-suppression methods, and the criteria used to define positivity (including variant allele frequency cutoffs and the number of required loci). This heterogeneity reduces inter-laboratory comparability, complicates the definition of universal thresholds, and limits the feasibility of robust cross-study meta-analyses (52, 89).

In addition, ctDNA assays are vulnerable to confounding by non-tumor sources of somatic mutations, most notably clonal hematopoiesis of indeterminate potential (CHIP). Mutations in genes such as TP53, DNMT3A, and TET2 are common, particularly in older individuals and smokers. If matched leukocyte DNA is not sequenced in parallel, CHIP-derived variants may be misclassified as tumor-associated, leading to overestimation of mutational burden or false-positive MRD calls (49). Dynamic metrics based on changes in variant allele frequency, including parameters such as mean mutant variant allele frequency and MinerVa-Delta, are also influenced by sequencing depth, panel design, and background error rates, which makes it difficult to directly extrapolate thresholds defined in one study to other platforms or centers.

### 4.2 Tumor biology and disease spectrum limitations

ctDNA reflects the component of tumor burden that sheds DNA into the bloodstream rather than the entire disease burden. Shedding efficiency varies across anatomical sites. Brain metastases, isolated pleural lesions, or residual locoregional disease may release DNA predominantly into compartments such as cerebrospinal fluid or pleural effusion, with limited contribution to plasma ctDNA. In these sanctuary sites, blood-based ctDNA may therefore underestimate the true extent of disease (49).

In lung cancer, this limitation is particularly important when considering de-escalation of immunotherapy on the basis of ctDNA negativity. Studies such as MERMAID-1 and MERMAID-2, the work by Moding et al., and BTCRC LUN 16–081 consistently show that patients with negative ctDNA after surgery or CRT have a better prognosis than ctDNA-positive patients, but a proportion of ctDNA-negative patients still experience late recurrence or distant metastasis. Thus, ctDNA negativity should be interpreted as lower risk rather than complete safety.

Similarly, although early ctDNA dynamics often correlate with immunotherapy response or resistance earlier than imaging, they are not perfectly concordant with long-term outcomes. In an NSCLC cohort summarized by Stadler et al., early ctDNA kinetics were discordant with eventual clinical benefit in about 20% to 25% of patients, indicating that longer follow-up and multi-timepoint monitoring are needed to improve response classification and prognostic robustness (90). In immunotherapy-specific scenarios such as pseudoprogression and hyperprogression, ctDNA can provide helpful adjunctive information, but it cannot fully replace integrated radiologic and clinical assessment.

### 4.3 Insufficient evidence level and clinical utility

Most available evidence characterizes ctDNA either as prognostic or as response-associated within treated cohorts. Many studies described as ‘predictive’ are retrospective or prospective exploratory cohorts, often with small samples and single-arm, single-center designs; such designs cannot establish treatment-specific benefit without an appropriate comparator and interaction analysis. Representative examples include the chemoradiotherapy (CRT) cohorts with or without consolidation ICI reported by Moding et al., the CRT cohort examined by Pan et al., the prospective cohort described by Yang et al., and ctDNA substudies embedded within trials such as BR.36 or those evaluating MinerVa-Delta. By contrast, truly prospective randomized trials that explicitly use ctDNA to guide whether immunotherapy should be given, and for how long, remain uncommon. As a result, the literature to date has largely established clinical validity rather than clinical utility. To show that ctDNA-guided care improves outcomes, prospective interventional studies are still needed. These trials should treat ctDNA as an actionable decision tool and prespecify algorithms that connect defined ctDNA states to treatment escalation, de-escalation, or therapy switching (6, 91).

### 4.4 Practical application and regulatory hurdles

In routine practice, several practical barriers continue to limit ctDNA implementation, including cost, turnaround time, and reimbursement. High-depth sequencing with personalized, tumor-informed panels can achieve excellent sensitivity and specificity, yet it depends on adequate tumor tissue, longer processing workflows, and specialized bioinformatics capacity, which together restrict broad deployment (92). At the same time, variability across commercial assays, such as differences in panel design, variant annotation, and reporting formats, makes cross-platform interpretation difficult and can leave clinicians uncertain about how to act on results. International expert groups have therefore emphasized standardization of testing workflows, rigorous validation of analytical performance, and clearer regulatory pathways as priorities for accelerating translation of liquid biopsy technologies (93). Additional system-level challenges include establishing multicenter quality control, securing insurance coverage, and integrating ctDNA-driven strategies into national and international guidelines.

These barriers do not negate the consistent prognostic and response-associated signals summarized in this review; rather, they define the boundary of ctDNA’s current role. ctDNA can presently support research-grade risk stratification and response assessment, whereas treatment selection, escalation, de-escalation, and duration remain investigational applications. Translation into routine lung cancer care will require harmonized technologies and analytical pipelines, prospective ctDNA-driven interventional trials, and clear implementation and regulatory pathways.

## 5 Summary and future perspectives

This review synthesizes evidence for ctDNA use in lung cancer immunotherapy across neoadjuvant, adjuvant, consolidation, and advanced-stage settings. Postoperative MRD, locally advanced NSCLC, advanced-disease cohorts, and limited-stage small-cell lung cancer (SCLC) data converge on a consistent conclusion: ctDNA is strongly supported as a prognostic and response-associated biomarker, whereas its ability to identify differential benefit from a specific immunotherapy remains investigational.

From a clinical standpoint, ctDNA appears to add value in three connected ways. First, MRD assessment after definitive local therapy, whether surgery or chemoradiation, can separate higher-risk from lower-risk patients and help frame decisions around adjuvant or consolidation immunotherapy. Second, early on-treatment ctDNA kinetics can provide a molecular readout of efficacy ahead of radiographic change, which is particularly helpful when trying to distinguish true benefit from primary resistance. Third, sustained ctDNA negativity during follow-up tends to align with durable remission and, at least in principle, could support carefully considered de-escalation to reduce toxicity and cost. At present, most of these observations remain correlational, but they offer a practical framework for personalization (85).

The next step is to move beyond association and demonstrate clinical utility. Prospective interventional trials should test ctDNA-guided algorithms, such as randomizing MRD-positive patients to intensified strategies or evaluating treatment pauses in patients with prolonged molecular remission, while also incorporating standardized methods and health economic assessments to support broader adoption (94).

On the technical side, ctDNA will likely evolve from a standalone marker to one component within multi-omics models, since immunotherapy response reflects complex interactions between the tumor and the host immune system (50, 95). Integrating quantitative ctDNA trends with blood-based TMB, resistance alterations, and immune signatures may better capture benefit and toxicity risk (59, 96). Implementation will also require optimized sampling and surveillance, including context-specific fluids such as CSF or pleural effusions, and, at the system level, harmonized laboratory standards, clear reporting conventions, and feasible reimbursement pathways (97). For now, ctDNA is best viewed as an adjunctive decision aid that is powerful for risk refinement and promising for prediction, until ongoing ctDNA-guided trials provide definitive evidence for routine, decision-defining use (98–100).
